# Levels of stunting associated factors among under-five children in Ethiopia: A multi-level ordinal logistic regression analysis

**DOI:** 10.1371/journal.pone.0296451

**Published:** 2024-01-02

**Authors:** Yordanos Sisay Asgedom, Beminate Lemma Seifu, Kusse Urmale Mare, Zufan Alamire Asmare, Hiwot Altaye Asebe, Bizunesh Fantahun Kase, Abdu Hailu Shibeshi, Tsion Mulat Tebeje, Kebede Gemeda Sabo, Bezawit Melaku Fente, Gizachew Ambaw Kassie, Afework Alemu Lombebo

**Affiliations:** 1 Department of Epidemiology, College of Health Sciences and Medicine, Wolaita Sodo University, Sodo, Ethiopia; 2 Department of Public Health, College of Medicine and Health Science, Samara University, Afar, Ethiopia; 3 Department of Nursing, College of Medicine and Health Sciences, Samara University, Afar, Ethiopia; 4 Department of Ophthalmology, School of Medicine and Health Sciences, Debre Tabor University, Debre Tabor, Ethiopia; 5 Department Statistics, College of Natural and Computational Science, Samara University, Afar, Ethiopia; 6 School of Public Health, College of Health Science and Medicine, Dilla University, Dilla, Ethiopia; 7 Department of General Midwifery, School of Midwifery, College of Medicine & Health Sciences, University of Gondar, Gondar, Ethiopia; 8 School of Medicine, College of Health Sciences and Medicine, Wolaita Sodo University, Sodo, Ethiopia; Debre Berhan University, ETHIOPIA

## Abstract

**Introduction:**

Stunting is a major public health problem affecting more than one-third of under five year’s old children in Ethiopia. It has short and long (irreversible) consequences, including stunted growth, never reaching physical and cognitive potential, struggles in school, and increased morbidity and mortality due to infections. Though stunting is the leading cause of child mortality in Ethiopia, evidence is scarce on the prevalence and predictors of stunting among under-five years old children in Ethiopia. Therefore, this study aimed to estimate the prevalence and predictors of stunting severity among under-5 children in Ethiopia.

**Materials and methods:**

This study was based on 2019 Mini-Ethiopian Demographic and Health Survey (EDHS) data. A weighted total sample of 4972 under-five years old children was included in the study. Height measurement was collected for each child. Anthropometric indicator, height-for-age was determined for children using World Health Organization growth standards (Z-scores for Height-for-Age (HAZ)) to asses stunting level. Given the ordinal nature of stunting and the hierarchical nature of EDHS data, a multilevel ordinal logistic regression model was applied. Brant test was used to check the proportional odds assumption, which was satisfied (P-value ≥0.05). Moreover, deviance was used for model comparison. For the multivariable analysis, variables with a p-value ≤0.2 in the bivariable analysis were considered. The Adjusted Odds Ratio (AOR) with 95% Confidence Interval (CI) was reported as associated factor to the severity levels of stunting in the multivariable multilevel proportional odds model.

**Results:**

The overall prevalence of stunting among under-5 children in Ethiopia was 35.7% [95% CI: 34.4%, 37.1%]. Of these, 12.1% were severely stunted, and 24.9% were moderately stunted. Being male [AOR = 0.83, 95% CI: 0.74, 0.93], children aged 6–23 months [AOR = 2.38, 95% CI: 1.84, 3.07], ≥ 24 months [AOR = 4.15, 95% CI: 3.26, 5.28], children whose maternal age 15–24 years [AOR = 0.73, 95% CI: 0.58, 0.92], children from the poorest, poorer, middle, and richer household wealth were [AOR = 1.84, 95% CI: 1.32, 2.57], [AOR = 1.66, 95% CI: 1.20, 2.31], [AOR = 1.78, 95% CI: 1.29, 2.44], and [AOR = 1.62, 95% CI: 1.20, 2.17], children whose maternal educational status of no formal education and primary education had [AOR = 1.90, 95% CI: 1.28, 2.82], [AOR = 1.78, 95% CI: 1.22, 2.60], Tigray [AOR = 2.95, 95% CI: 1.78, 4.86], Afar [AOR = 1.85, 95% CI: 1.11, 3.10], Amhara [AOR = 1.90, 95% CI: 1.14, 3.14] and Harari [AOR = 1.97, 95% CI: 1.20, 3.25]regions, low community maternal education [AOR = 0.76, 95% CI: 0.62, 0.92] were significantly associated with stunting severity levelling.

**Conclusion:**

Stunting among children under five years of old in Ethiopia remains a major public health issue. Improving access to maternal education is related to appropriate child feeding practices and health, particularly in younger and uneducated mothers. Strengthening the family’s wealth status is also recommended to reduce stunting. In addition, it is better to support strategies of preconception care for mothers during pregnancy to reduce stunting in the long term.

## Introduction

Stunting is characterized by low height-for-age, which hinders children’s ability to reach their full physical and cognitive potential due to persistent or repeated under nutrition [1]. Stunting is best predicted by length and height, as having a length/height ratio that is more than two standard deviations below the population’s median [2]. The impact of the disease is serious and long-lasting, for children, families, communities, and the country as a whole.

Stunting affected (too short for age) an estimated 149.2 million children under 5 globally in 2020, with variation across different sub-regions of the world based on geographical location [1, 3]. In 2020, more than half (53%) of under-five years old children were affected by stunting lived in Asia, and two out of five or more than 41% lived in Africa [4]. The number of children with stunting has declined in the past two decades (from to 2000–2020) in all regions, except Africa and Asia [4].

Based on the 2019 Mini Ethiopian Demographic and Health Survey, the prevalence of stunting in Ethiopia is 37%, indicating that stunting is still a major public health issue in the country [5]. It has devastating consequences that can last for a lifetime and even affect the next generation. Stunted children may never reach their full height potential and their brains may never reach their full cognitive potential [4].These children start their lives at a significant disadvantage: they struggle in school, earn less as adults, and face barriers to community participation [4]. Moreover, stunted children are at a greater risk of infectious morbidity and mortality [6].

Stunting is usually associated with poor socioeconomic conditions, poor maternal health and nutrition, frequent illness, and inappropriate infant and young child feeding and care in early life [1]. Previous research has identified various factors that are associated with stunted growth in children. In particular, several independent predictors of stunting have been identified in Ethiopia, including the child’s sex, age, household wealth status, the interval between births, place of residence, the mother’s educational attainment, maternal height, body mass index (BMI), region, religion, contraceptive use, and mental health status [7–15].

The World Health Organization (WHO) aims to reduce the prevalence of stunting by 40% by 2025 [16]. About 37% of children under five years old in Ethiopia currently affected by stunting [4]. In addition, the Ethiopian government has pledged to end child under nutrition, particularly stunting, by 2030 [17]. In Ethiopia, studies on the prevalence of stunting and its associated factors in children under five years old are being conducted. However, these studies cannot comprehend the ordinal nature of stunting status because the impact of stunting varies depending on the severity level of stunting (mild, moderate, and severe stunting). Consequently, we used multilevel ordinal logistic regression to obtain a reliable estimate while avoiding information loss. Thus, the present study aimed to assess the levels of stunting-associated factors among under-five children in Ethiopia using Mini-EDHS 2019 data.

## Materials and methods

### Data source and sampling procedure

This study was based on the 2019 mini-DHS of Ethiopia. The EDHS is conducted every five years to generate updated health and health-related indicators. The data were derived from the measure DHS (Demographic and Health Survey) program and detailed information about the surveys can be found in each country’s DHS reports. A multistage stratified sampling technique was employed to select the study subjects. In the first stage, Enumeration Areas (EAs) were randomly selected, whereas in the second stage, households were selected. There are different datasets in DHS, and for this study, we used the Kids Record (KR) file. The dependent and independent variables were extracted from the KR dataset, based on the literature. The final sample size was 4,972.

### Study variables and measurements

#### Dependent variable

Our outcome variable of interest was levels of stunting among under-five years old children, which we grouped into four ordinal categories; severely stunted if the Z-score < −3 Standard Deviation (SD), moderately stunted (−3.00 ≤ HAZ < 2); and not stunted if Z-score ≥ −2 SD.

#### Independent variables

Because of the hierarchical nature of the DHS data, the independent variables considered in the study were obtained from two sources (individual and community-level variables). Child age, child sex, household wealth status, maternal education, maternal age, sex of household head, child twin status, birth order, parity, number of Ante Natal Care (ANC) visits, place of delivery, husband’s education, type of toilet facility, type of water source, birth size, and maternal occupation were level one variables. Residence, region, community poverty, and community maternal education were level-two variables.

These two community-level variables (community maternal education and poverty) were generated by aggregating maternal education and household wealth status at the cluster/enumeration area levels. They were then categorized as having higher community maternal education and poverty based on the national median value of maternal education and poverty since they were not normally distributed.

### Data management and analysis

The data were weighted using sampling weight, primary sampling unit, and strata before any statistical analysis to restore the representativeness of the survey and take into account the sampling design when calculating standard errors, to obtain reliable statistical estimates. STATA (StataCorp, USA) version 17 statistical software was used for data management and analysis. The outcome variable for this study was polychotomous and had ordinal nature. Therefore, an ordinal logistic regression model was used to the analyze the predictors of stunting (not stunted, moderately, and severely stunted). The fundamental assumption in an ordinal logistic regression model is proportional odds. If the data satisfies the proportional odds assumption, the proportional odds model can be used; otherwise, a partial proportional model should be used. To choose the appropriate ordinal model for the data, we checked the Proportional Odds (PO) assumptions, which state that the effects of all independent variables are constant across categories of the outcome variable. After fitting the proportional odds model, the proportional odds assumption was tested using the Brant’s test. It tests the null hypothesis that there is no difference in the effects of the independent variables across the stunting levels. The Brant test revealed that the proportional odds assumption was satisfied (p>0.05). Therefore, we used the proportional odds model to assess predictors of stunting levels and independent variables.

Besides, DHS data have a hierarchical nature. Therefore, children and mothers are nested within a cluster, and we assume that study subjects in the same cluster may share similar characteristics with participants in another cluster. This violates the independence observations and equal variance assumptions between the clusters in the ordinal logistic regression model. This implies the need to consider heterogeneity between clusters using an advanced model. Therefore, a multilevel cumulative logit model was used.

As a result, because the Brant test was met, the multilevel proportional odds model gave a single Odds Ratio (OR) for an explanatory variable (severe vs. moderate/not stunted, severe/moderate vs. not stunted).

Four models were constructed for multilevel logistic regression analysis. The first model was a null model without explanatory variables to determine the extent of cluster variation in stunting levels. The second model was adjusted for individual-level variables; the third model was adjusted for community-level variables, and the fourth was fitted with both individual and community-level variables simultaneously. Model comparison was made based on deviance (-2Log-Likelihood Ratio (LLR)) because the models were nested models, and the model with the lowest deviance was the best-fitted model for the data.

Variables with a p-value ≤ 0.2 in the bi-variable multilevel proportional odds model were considered for the multivariable multilevel proportional odds model. In the multivariable multilevel proportional odds model, the Adjusted Odds Ratio (AOR) with 95% Confidence Interval (CI) was reported to indicate the strength of association, and the statistical significance for the final model was set at p<0.05.

## Results

### Study participant’s descriptive characteristics

A total of 4,972 children under five years old were included (Table 1). Of these, 2967 (59.66%) were children aged ≥24 months and 2530 (50.89%) were male. About 2527 (50.82%) were from parity ≥4, 1702 (34.23%) were from parity 2–3 and 4864 (97.83%) type of birth were single. Nearly half (51.02%) of the mothers gave birth at home, and 1575 (43.66%) attended ≥4 ANC visits. More than half, 2665 (53.60%) mothers did not attain formal education and 1146 (23.05%) were aged≥ 35. Regarding household wealth status, around 1,141 (22.94%) were the poorest and 4329 (87.07%) heads of households were males. About 2708 (54.46%) > 5 household numbers and 4,169 (83.85%) types of toilet facilities did not improve. The majority (64.99%) were available for to an improved water source and 4688 (94.28%) were married.

**Table 1:** Individual-level characteristics of the study participants in Ethiopia.

Variable	Weighted frequency	Percentage (%)
**Child age in months**		
<6	529	10.64
6–23	1476	29.7
≥24	2967	59.66
**Parity**		
One	743	14.95
2–3	1702	34.23
≥4	2527	50.82
**Sex of child**		
Male	2530	50.89
Female	2442	49.11
**Type of birth**		
Single	4864	97.83
Multiple	108	2.17
**Place of delivery**		
Home	2537	51.02
Health facility	2435	48.98
**Number of ANC visits (n = 3608)**		
No	891	24.68
1–3	1142	31.66
≥4	1575	43.66
**Maternal educational status**		
No education	2665	53.60
Primary	1759	35.39
Secondary	366	7.37
Higher	181	3.65
**Household wealth status**		
Poorest	1141	22.94
Poorer	1098	22.09
Middle	934	18.78
Richer	881	17.72
Richest	918	18.46
**Sex of household head**		
Male	4329	87.07
Female	643	12.93
**Number of household members**		
≤5	2264	45.54
>5	2708	54.46
**Type of toilet facility**		
Improved	803	16.15
Not improved	4169	83.85
**Availability of improved water source**		
Improved	3231	64.99
Not improved	1741	35.01
**Maternal age in years**		
15–24	1139	22.92
25–34	2687	54.04
≥35	1146	23.05
**Marital status**		
Not married/divorced/widowed	284	5.72
Married	4688	94.28

Regarding the community-level characteristics of the study participants, 3723 (74.88%) were from rural areas, 1979 (39.80%) were from the Oromia region, 1008 (20.27%) were from Southern Nation, Nationalities and People Representatives (SNNPRs), and 14(0.29%) were from Harar. Community maternal education was low in nearly half (51.37%) of the study participants, and the level of poverty was high in 2755 (55.41%) of the community (Table 2).

**Table 2:** Community-level characteristics of the study participants in Ethiopia.

Variables	Weighted frequency	Percentage (%)
**Residence**		
Urban	1249	25.12
Rural	3723	74.88
**Region**		
Tigray	351	7.06
Afar	75	1.50
Amhara	957	27.81
Oromia	1979	39.80
Somali	344	6.91
Benishangul-gumuz	59	1.18
SNNPRs	1008	20.27
Gambella	21	0.43
Harar	14	0.29
Dire-Dawa	26	0.52
Addis Ababa	138	2.78
**Community maternal education**		
Low	2554	51.37
High	2418	48.63
**Community poverty**		
Low	2217	44.59
High	2755	55.41

**SNNPRs:** Southern Nation, Nationalities and People Representatives

#### Prevalence of levels of stunting

The overall prevalence of stunting among under five years old children was 35.7% [95% CI: 34.4%, 37.1%]. This study showed that 24.9% [95% CI: 23.5%, 25.9%] of under five years old children had moderate stunting, and 12.1% [95% CI: 11.2%, 13.0%] had severe stunting.

#### Random effect analysis results

*Null model (Model 1)*. This model is an intercept-only model without predictors. We examined whether the multilevel ordinal logistic regression model was more significant over the single-level ordinal logistic regression model using the Likelihood Ratio (LR). The LR test result was statistically significant (p<0.05), indicating that the multilevel ordinal logistic regression model was best fitted to the single-level ordinal logistic regression analysis. Therefore, the LR-test was suggestive of use of a multilevel ordinal logistic regression model. Four random effect models were fitted and the final model was chosen since it had the lowest deviance value (Table 3).

**Table 3:** Multilevel ordinal logistic regression analysis of factors associated with levels of stunting among under-five children in Ethiopia.

Variables	Null model	Model I (with level one variables)	Model II (with level II variables)	Model III (with level 1 and level 2 variables)
**Sex of child**				
Male		1		1
Female		0.84 (0.74, 0.94)		0.83 (0.74, 0.93)*
**Child age in months**				
<6		1		1
6–23		2.36 (1.82, 2.57)		2.38 (1.84, 3.07)*
≥24		3.50 (3.37, 3.64)		4.15 (3.26, 5.28)*
**Family size**				
≤5		1		1
>5		1.09 (0.92, 1.30)		1.11 (0.94, 1.32)
**Parity**				
One		1		1
2–3		0.85 (0.69, 1.04)		0.85 (0.69, 1.05)
≥4		0.88 (0.67, 1.14)		0.90 (0.69, 1.18)
**Maternal age**				
15–24		1		1
25–34		0.93 (0.78, 1.11)		0.93 (0.78, 1.12)
≥35		0.73 (0.58, 0.92)		0.73 (0.58, 0.92)*
**Marital status**				
Married		1.25 (0.95, 1.64)		1.16 (0.88, 1.53)
Others		1		1
**Place of delivery**				
Health facility		1.11 (0.96, 1.29)		1.12 (0.97, 1.30)
Home		1		^1^
**Household wealth status**				
Poorest		2.26 (1.76, 2.91)		1.84 (1.32, 2.57)*
Poorer		2.07 (1.61, 2.67)		1.66 (1.20, 2.31)*
Middle		2.21 (1.71, 2.85)		1.78 (1.29, 2.44)*
Richer		1.89 (1.47, 2.42)		1.62 (1.20, 2.17)*
Richest		1		1
**Maternal education status**				
No education		2.18 (1.47, 3.23)		1.90 (1.28, 2.82)*
Primary		1.88 (1.28, 2.76)		1.78 (1.22, 2.60)*
Secondary		1.14 (0.74, 1.75)		1.13 (0.74, 1.73)
Higher		1		1
**Residence**				
Urban			1	1
Rural			1.33 (1.05, 1.69)	0.99 (0.76, 1.31)
**Region**				
Addis Ababa			1	1
Tigray			3.38 (2.07, 5.54)	2.95 (1.78, 4.86)*
Afar			2.34 (1.42, 3.85)	1.85 (1.11, 3.10)*
Amhara			2.31 (1.41, 3.79)	1.90 (1.14, 3.14)*
Oromia			1.94 (1.20, 3.14)	1.51 (0.91, 2.48)
Somali			1.14 (0.68, 1.90)	0.85 (0.50, 1.44)
Benishangul-gumuz			2.16 (1.30, 3.57)	1.67 (0.99, 2.81)
SNNPR			2.06 (1.26, 3.34)	1.61 (0.97, 2.65)
Gambella			0.87 (0.51, 1.49)	0.71 (0.41, 1.22)
Harari			2.18 (1.34, 3.57)	1.97 (1.20, 3.25)*
Dire-Dawa			1.32 (0.79, 2.19)	1.19 (0.71, 1.99)
**Community poverty**				
Low			1	1
High			1.38 (1.13, 1.68)	1.17 (0.94, 1.47)
**Community maternal education**				
Low			1	1
High			0.68 (0.57, 0.83)	0.76 (0.62, 0.92)**
**Random effect analysis results**				
LLR	-4409.564	-4248.281	-4327.957	-4202.393
Deviance (-2LLR)	8819.128	8496.562	8655.914	8404.786
AIC	8825.128	8536.561	8687.874	8470.786
BIC	8844.725	8667.207	8792.391	8686.351
LR-test	LR test vs. logit model: chibar2(01) = 36.16 Prob > = chibar2 <0.001			

** AIC: Akaike Information Criteria, AOR: Adjusted Odds Ratio, BIC: Bayesian Information Criteria, LLR: Log-Likelihood Ratio, LR: Likelihood Ratio

#### Proportional odds assumption

The parallel-lines assumption was checked using the Brant test. The null hypothesis in the proportional odds assumption is that there is no difference in the effects of the independent variables across levels of stunting. The Brant test revealed that the proportional odds assumption was fulfilled (p = 0.21). In addition to the global test, we assessed each variable in the model to identify the variables for which the proportional odds assumption was fulfilled, and all the variables had a p-value>0.05.

#### Factors associated with levels of childhood stunting

Bivariable analysis was performed to identify the factors associated with stunting. Consequently, the sex of the child, child’s age in months, family size, parity, maternal age, marital status, place of delivery, household wealth status, maternal education status, residence, region, community poverty, and community maternal education were considered for multivariable analysis (p<0.2). In the multivariable multilevel proportional odds model; the sex of a child, maternal age, and community maternal education were significantly associated with lower odds of severity levels of stunting. However, child age in months, household wealth status, maternal education, and region were significantly associated with higher odds of stunting severity levels. The odds of having higher levels of stunting among female children were decreased by 17% [AOR = 0.83, 95% CI: 0.74, 0.93] compared to male children. The odds of having higher levels of stunting among children aged 6–23 months and ≥ 24 months were 2.38 times [AOR = 2.38, 95% CI: 1.84, 3.07] and 4.15 times [AOR = 4.15, 95% CI: 3.26, 5.28] higher odds compared to children aged < 6 months. Children whose maternal aged ≥ 35 years their odds of having higher levels of stunting decreased by 27% [AOR = 0.73, 95% CI: 0.58, 0.92] compared to those whose mothers aged 15–24 years. Children from the poorest, poorer, middle, and richer household wealth were 1.84 times [AOR = 1.84, 95% CI: 1.32, 2.57], 1.66 times [AOR = 1.66, 95% CI: 1.20, 2.31], 1.78 times [AOR = 1.78, 95% CI: 1.29, 2.44], and 1.62 times [AOR = 1.62, 95% CI: 1.20, 2.17] higher odds of higher level of stunting compared to children from the richest household wealth, respectively. Children whose maternal educational status of no formal education and primary education had 1.90 times [AOR = 1.90, 95% CI: 1.28, 2.82], 1.78 times [AOR = 1.78, 95% CI: 1.22, 2.60] higher odds of a higher level of stunting than children whose mother had a higher level of education, respectively.

The odds of being at higher levels of stunting among children in Tigray, Afar, Amhara and Harari regions were 2.95 times [AOR = 2.95, 95% CI: 1.78, 4.86], 1.85 times [AOR = 1.85, 95% CI: 1.11, 3.10], 1.90 times [AOR = 1.90, 95% CI: 1.14, 3.14] and 1.97 times [AOR = 1.97, 95% CI: 1.20, 3.25] compared to children in Addis Ababa. The Odds of having higher levels of stunting among children from high community maternal education decreased by 24% [AOR = 0.76, 95% CI: 0.62, 0.92] compared to children from low community maternal education (Table 3).

## Discussion

The prevalence of stunting in Ethiopia was 35.77% [95% CI: 34.4%, 37.1%], indicating that childhood stunting remains a major public health problem in Ethiopia. Although the government of Ethiopia is committed to accelerating the reduction of stunting as a key strategy for human capital development and inclusive economic development, stunting remains a serious public health issue in the country. It is higher than the prevalence reported in Northern Brazil (14.8) [18], Pakistan (21%), and China (20%) [19]. The potential reason may be due to differences in socioeconomic status among countries; China and Brazil have a higher socioeconomic status than Ethiopia [20]. It is also possible that countries with a middle or high socioeconomic status have better chance of combating nutritional issues such as stunting by providing appropriate nutritional supply and variety to their populations than low-income countries such as Ethiopia [21]. Moreover, in Ethiopia, there is a widespread belief that young children are unable to chew and digest meat and dairy products and, as a result, are not provided with these foods, thereby missing out on important proteins and micronutrients that favour the occurrence of stunting [22].

Further, in the final model, we found that the sex of the child, maternal age, and community maternal education were significantly associated with lower odds of stunting severity levels, whereas child age in months, household wealth status, maternal education, and region were significantly associated with higher odds of stunting severity level.

Being male was significantly associated with increased odds of childhood stunting compared to being female. This is in line with studies conducted in Tanzania [23], Indonesia [7], and Nigeria [24]. This may because nutrition and health tend to be worse in boys beginning in utero and continuing through childhood [25]. Another reason might be that boys are more likely to be born preterm and have low birth weight, which could also contribute to stunting [25, 26]. Children aged 6–23 months and above had higher odds of higher levels of stunting than children aged less than 6 months. A possible reason might be that the age of 6–23 months is the crucial time to begin complementary feeding, which exposes children to frequent infections, further causing stunting in older children [27].

Children born to mothers aged 15–24 had higher odds of higher levels of stunting than children born to mothers aged 25 years and above. This could be because babies born to younger mothers are more likely to be preterm and have low birth weight, which may predispose the newborn to neonatal infections and malnutrition such as stunting [28]. Moreover, younger mothers are less able to ensure adequate nutritional intake for their children since the mother is still in the growing stage, which affects their children’s growth and development, resulting in malnutrition and other growth impediments [29].

Children from families with low household wealth had increased odds of having higher levels of stunting than those children from rich households. This is in line with findings reported from Oromia [30], Indonesia [7], Nigeria [31], the Democratic Republic of Congo [32], and Rwanda [12]. The possible reason might be low household wealth status is associated with food insecurity which will not allow them to be well nourished and more predisposed them to growth failure. Further, the high prevalence of stunting might be because households with low wealth status are less likely to have sufficient nutrient-rich purchases and access to healthcare services during their children illness.

Poor maternal educational status was significantly associated with increased odds of childhood stunting. This is consistent with the findings reported in previous studies [3, 12, 18, 28]. A possible explanation might be that maternal education is a reliable predictor of nutritional outcomes [33, 34]. Maternal education helps to improve the understanding of infant health and nutrition (e.g., exclusive breastfeeding and appropriate complementary feeding), which helps to improve the quality of children’s diets [35]. Moreover, higher maternal education is associated with greater healthcare-seeking behaviour for their children than their counterparts [36].

Children from Tigray, Afar, Amhara, and Harari regions were significantly associated with higher odds of having higher levels of stunting compared to Addis Ababa. This is consistent with the studies reported in [37] and, Nigeria [24]. A possible reason might be the availability and accessibility of education, information on how to care for their children, and health care services.

Children who lived in a community with a high level of maternal education had lower odds of higher levels of stunting compared to children from low community maternal education. This is consistent with another study [38]. This is because low community maternal education (illiteracy) is the principal cause of childhood malnutrition [39]. Further, in contrast to an illiterate society, educated communities are more knowledgeable about possible childcare practices, have better nurturing, use healthcare services, and have more independence in making decisions [40, 41].

Public health and methodological implications have been looked at in this study. In terms of public health, the study was based on a large sample size of Ethiopian children using pooled Mini-EDHS data, increasing the statistical power of the study and allowing the findings to be generalized. In terms of methodology, previous research has employed binary classification of stunting using yes/no categories, but treating mild, moderate, and severe stunting as a yes is not statistically valid as it leads to the loss of crucial information. Additionally, the factors responsible for mild stunting may not be the same as those responsible for severe stunting, highlighting the need for more nuanced analysis.

This study had both strengths and limitations. The study utilized a weighted pooled nationally representative EDHS survey in Ethiopia, and multilevel ordinal logistic regression analysis was conducted to obtain reliable estimates and standard errors. The sample size was sufficient to detect the true effects of the independent variables. However, readers should keep in mind the limitations of the study, as it cannot establish a causal relationship between stunting and predictors due to the use of cross-sectional data. Additionally, the study was not able to explore all variables important to childhood stunting as it relied on secondary data.

## Conclusions

Stunting among under five years old children in Ethiopia is a key public health problem. The sex of the child, child age in months, maternal age, household wealth status, maternal educational status, region, and community maternal education were found to be significant predictors of the severity levels of stunting. Improving access to maternal education related to appropriate child-feeding practices and health, particularly in younger and uneducated mothers. Strengthening the wealth status of the family is also recommended to decrease stunting. Besides, it is better to support strategies of preconception care for mothers during pregnancy to reduce stunting in the long term.

## Abbreviations

AOR: Adjusted Odds Ratio
ANC: Antenatal care
DHS: Demographic and Health Survey
EDHS: Ethiopian Demographic health survey
CI: Confidence Interval
EAs: Enumeration areas
ICC: Intra-cluster Correlation Coefficient
JME: Joint Malnutrition Estimates
HAZ: Z-score for Height-for-Age
KR: Kids Record
LLR: Log likelihood ratio
LR: Likelihood ratio
OR: Odds ratio
SDG: Sustainable Development Goal
SSA: sub-Saharan Africa
SNNPRs: Southern Nation, Nationalities and People Representatives WHO = World Health Organization
WHA: World Health Assembly
